# Juvenile Alexander Disease: A Rare Leukodystrophy

**DOI:** 10.7759/cureus.24870

**Published:** 2022-05-10

**Authors:** Rizwan Ullah, Muhammad Hayyan Wazir, Aiysha Gul, Faiza Gul, Amna Arshad

**Affiliations:** 1 Internal Medicine, Hayatabad Medical Complex, Peshawar, PAK; 2 Obstetrics and Gynecology, Mardan Medical Complex, Mardan, PAK

**Keywords:** leukodystrophy, rare, disease, alexander, juvenile

## Abstract

Alexander disease is an uncommon autosomal dominant leukodystrophy that influences the white matter of the central nervous system (CNS), predominantly affecting the frontal lobe bilaterally. The most obvious pathogenic hallmark is the extensive deposition of cytoplasmic inclusions known as "Rosenthal fibers" in perivascular, subpial, and subependymal astrocytes throughout the CNS. The hereditary cause is mutations in the glial fibrillary acidic protein (GFAP) gene. Infantile, adult, and juvenile onsets are the three subtypes. Psychomotor retardation, mile-stone regression, spastic paresis, brain stem symptoms (swallowing, speech, etc.), and seizures define the juvenile variety, which emerges between the ages of three and 10 years. Macrocephaly has a lower likelihood of being a juvenile type. It is generally diagnosed based on clinical and magnetic resonance imaging findings. A five-year-old girl is presented as a case of juvenile Alexander disease, with typical clinical and MRI features.

## Introduction

Alexander disease (AD) is an uncommon, autosomal dominant leukodystrophy that influences the white matter of the central nervous system (CNS), with a predilection for the frontal lobe [1]. The far more evident pathogenic feature is the extensive deposition of cytoplasmic inclusions known as "Rosenthal fibers" throughout the central nervous system (CNS), primarily in subependymal astrocytes, subpial, and perivascular. Glial fibrillary acidic protein (GFAP) gene mutations are the genetic cause [2]. Infantile, juvenile, and adult forms are the three types of clinical subgroups. Around 51% of those affected are infants, 23% are juveniles, and 24% are adults [3]. During the first two years of life, the infantile type is most frequent, and children can live anywhere from a few days after birth to around the age of eight to 10 years. Macrocephaly and psychomotor delay are present in these children, which are followed by regression, spastic paraparesis, and feeding difficulties. The juvenile variant, which appears between the ages of three and 10, is less likely to be associated with microcephaly and has more noticeable brain-stem symptoms, such as speech and swallowing difficulties. It proceeds more slowly than the infantile type and does not always result in the same degree of impairment. Juvenile cases can last anywhere from 10 years or early adolescence to young adulthood, and in some circumstances, much longer. The adult-onset variant lacks macrocephaly has a waddling gait and frequently exhibits lower brain stem and upper spinal cord dysfunction [4,5]. A diagnostic technique for this condition is magnetic resonance imaging (MRI), which demonstrates significant cerebral white matter alterations with a frontal preponderance and comparative sparing of occipital and temporal white matter, as well as abnormalities of the basal ganglia and thalamus in certain individuals [6]. We describe a five-year-old child with juvenile Alexander disease who has typical clinical and MRI findings.

## Case presentation

**Figure 1:**
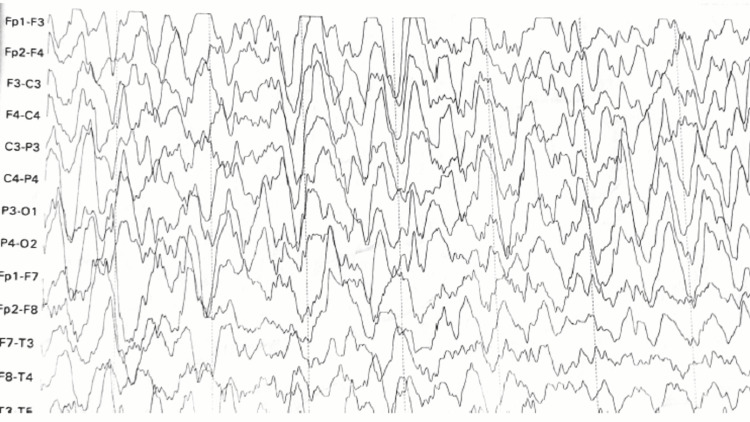
Abnormal EEG shows neurodegenerative changes.

A five-year-old girl presented to us with generalized fits. She has had three seizure episodes in the last four months. Her weight was 11 kg, with a normal head circumference for her age. The patient is the second child of non-consanguineous parents with no family history of neurological disorders. Normal birth and developmental history. She was fine until four months ago when she began to exhibit unusual social behavior (crying, shouting), and her developmental milestones began to regress (waddling gait, inability to sit, inability to hold neck, inability to talk, inability to understand). At the time of the first episode, she had started on valproic acid for the control of fits. On general physical examination, a child girl lying on a bed in a supine position with abnormal psychomotor behavior and regression was found. The head circumference was found to be normal. A neuromuscular examination showed signs and symptoms of pseudobulbar palsy, abnormal eye movements, and spastic quadriparesis. Extensive neurometabolic analysis, which included serum, urine, and cerebrospinal fluid, came up inconclusive. Electroencephalogram (EEG) shows mixed alpha and beta waves. There are bilaterally synchronously periodic generalized bursts of extremely high amplitude within a range of sharp and slow wave complexes with superimposed multifocal spikes and waves discharges (Figure 1).

**Figure 2:**
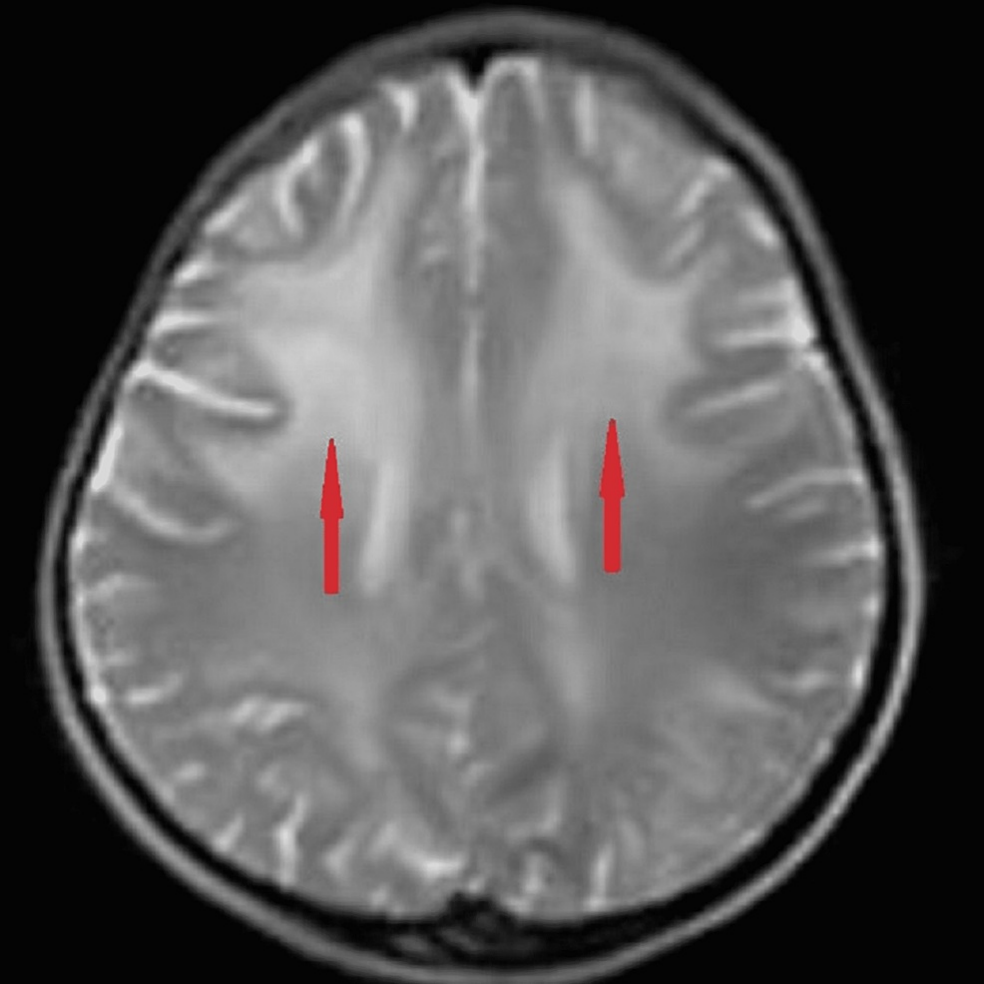
T2W image shows diffuse symmetrical, bilateral hyperintensities with a preponderance of frontal lobes (red arrows). T2W image: T2-weighted image

**Figure 3:**
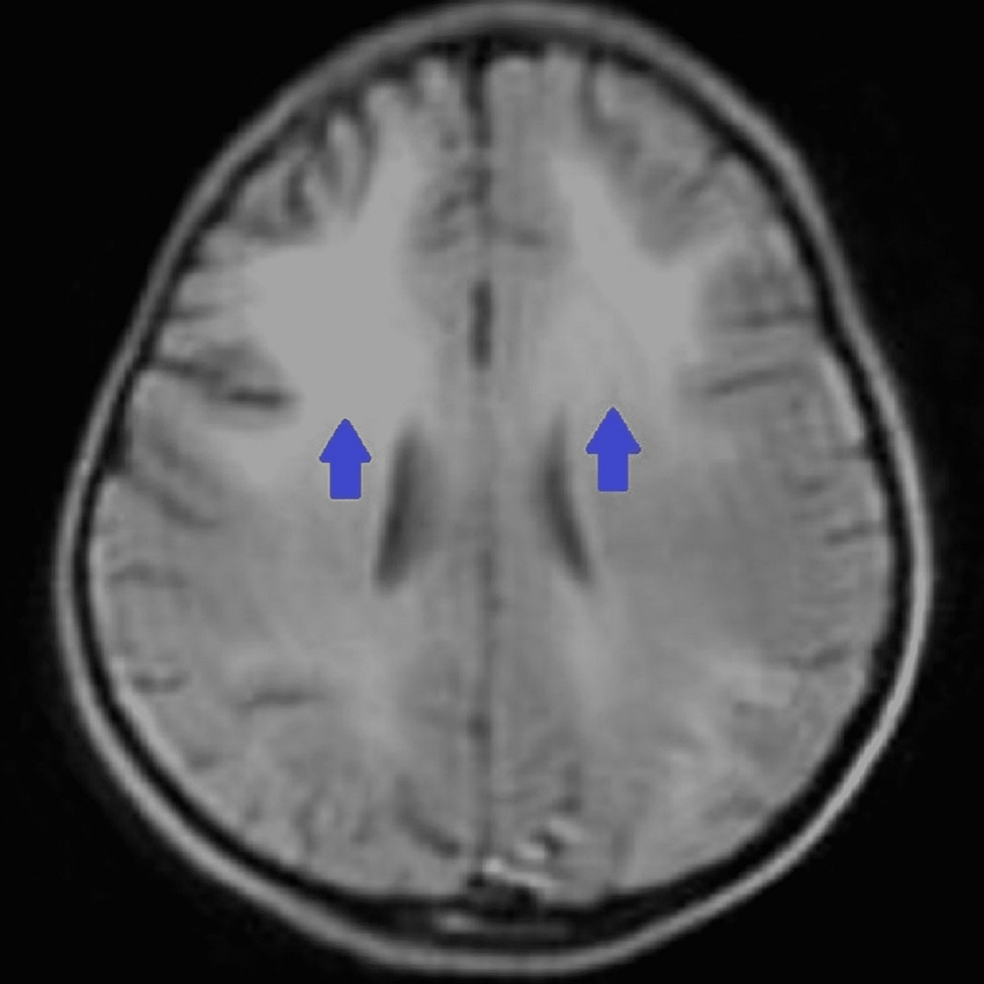
FLAIR image shows diffuse symmetrical, bilateral hyperintensities with preponderance of frontal lobes (blue arrows). FLAIR: fluid-attenuated inversion recovery

**Figure 4:**
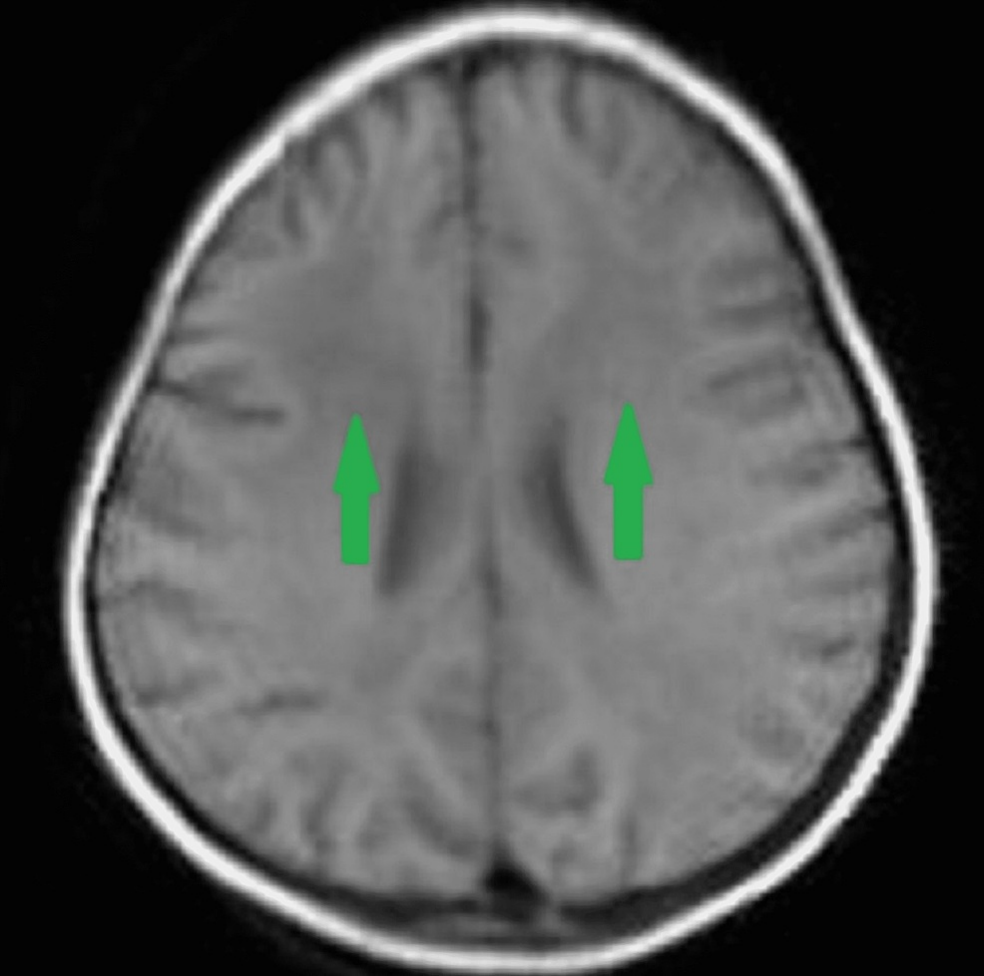
T1W image shows diffuse symmetrical, bilateral hypointensities with preponderance of frontal lobes (green arrows). T1W image: T1-weighted image

Magnetic resonance imaging (MRI) revealed bilateral, diffuse symmetrical, hyperintensities in the cerebral white matter involving periventricular areas and subcortical U-fibers with slight frontal lobe predominance on T2-weighted (T2W) images (Figure 2), fluid-attenuated inversion recovery (FLAIR) images (Figure 3), and hypointensities on T1-weighted (T1W) images in same distribution (Figure 4), no other positive findings. As we cannot perform brain biopsy and genetic testing are not available in our setup but these clinical, EEG, and MRI findings are suggestive of juvenile Alexander disease. We exclude Canavan disease and other leukodystrophies on the basis of bilateral, subcortical U-fibers involvement with frontal lobe predominance (diffuse in Canavan disease and no involvement in others); no ventriculomegaly and megalencephaly; and normal globi pallidi and thalami.

## Discussion

Alexander disease belongs to a group of neurological disorders which affect the white matter of the central nervous system, i.e., disrupted myelination of nerves (leukodystrophy) predominantly affects infants and children and can also affect adults [7]. Traditionally, Alexander disease was classified into Infantile, juvenile, and adult forms. However, revised classification is nowadays used, which has divided Alexander disease into two types. Type 1 is an early onset Alexander disease with predominant features - seizures, macrocephaly, motor and developmental delay, failure to thrive with paroxysmal deterioration, encephalopathy, and typical brain MRI findings suggestive of this disease. Type 2 presents across the life span with predominant features of eye movement abnormality, palatal clonus, bulbar involvement, autonomic dysfunction, and often cognitive and other neurological deficit with atypical brain imaging studies. Our case presented with seizures, abnormal eye movements, pseudobulbar signs, developmental milestone regression, and psychomotor retardations. Type 1 disease is more severe than type 2, with reported two-fold mortality seen in type 1 than in type 2 [8].

Mode of inheritance for Alexander disease showed an autosomal dominance pattern with mutation studied in GFAP gene acting as a gain of function mutation leading to disorder in dimerization of intermediate filaments resulting in accumulation of abnormal proteins and cytoskeleton collapse [9,10]. The disease's rarity was determined by a population-based study conducted in Japan, which estimated the prevalence of this disease to be one in every 2.7 million people. While a study in Germany reported that 1.6% of all leukodystrophies were diagnosed with Alexander disease [11].

To diagnose Alexander disease, clinical and radiological evidence should be established with genetic testing explicitly needed to know the presentation and heterogeneity of the disease. Confirmation is done by demonstrating the pathogenic variant of GFAP. In children with the recognized clinical feature of Alexander disease, the diagnosis can be strengthened by neuroimaging studies (brain MRI) which should fulfill the following diagnostic criteria - (1) abnormalities of the basal ganglia and thalami; (2) a periventricular rim of high signal on T1-weighted sequences and low signal on T2-weighted sequences; (3) widespread changes in white matter with frontal predominance; (4) abnormalities of the brain stem, involving the midbrain and medulla; and (5) contrast enhancement involves one or more of the following gray and white matter structures, i.e., dentate nucleus, optic chiasm, frontal white matter, periventricular tissue, fornix, basal ganglia, brain stem, ventricular lining, and thalamus. However, genetic testing is an effective tool in diagnosing children with atypical imaging features. Our case was diagnosed as juvenile Alexander disease based on the age of onset, and typical clinical and radiological features. Brain biopsy and genetics are not available in our setup. Neuroimaging criteria are least helpful for diagnosis of adult-onset Alexander disease [12].

Treatment of Alexander disease is supportive mainly with a multidisciplinary team, and a comprehensive team can enhance the affected person's quality of life [13]. Anti-seizure medication, proton-pump inhibitor (PPIs), antispasmodics, wheelchair for a non-ambulatory patient, bladder training, ventriculoperitoneal shunt in neonatal, or infantile obstructive hydrocephalus, etc. are used in these patient's management. It is reported that an adult with Alexander disease showed clinical improvement after prolonged use of intravenous ceftriaxone [14]. These patients show poor outcomes and depend on the specific form. The neonatal form has a worse prognosis, and most infantile form children do not live past the age of six. Furthermore, the juvenile and adult forms have a slower and longer course [15].

## Conclusions

Juvenile Alexander disease is a white matter neurodegenerative disorder characterized by seizure, spastic paresis, psychomotor retardation, milestone regression, and pseudobulbar signs. In this case study, we discussed a juvenile Alexander dystrophy with typical clinical and MRI findings. Most patients don't have all signs and symptoms of this disease but our patient has all the clinical features, EEG, and MRI findings of the disease which are helpful for the diagnosis of this rare disease.

## References

[1] Schmidt S, Wattjes MP, Gerding WM, van der Knaap M (2011). Late onset Alexander's disease presenting as cerebellar ataxia associated with a novel mutation in the GFAP gene. J Neurol.

[2] Matarese CA, Renaud DL (2008). Magnetic resonance imaging findings in Alexander disease. Pediatr Neurol.

[3] Srivastava S, Waldman A, Naidu S (2020). Alexander disease. GeneReviews [Internet].

[4] Mastri AR, Sung JH (1973). Diffuse Rosenthal fiber formation in the adult: a report of four cases. J Neuropathol Exp Neurol.

[5] Schwankhaus JD, Parisi JE, Gulledge WR, Chin L, Currier RD (1995). Hereditary adult-onset Alexander's disease with palatal myoclonus, spastic paraparesis, and cerebellar ataxia. Neurology.

[6] Pandit L (2009). Differential diagnosis of white matter diseases in the tropics: an overview. Ann Indian Acad Neurol.

[7] Barkovich AJ, Messing A (2006). Alexander disease: not just a leukodystrophy anymore. Neurology.

[8] Prust M, Wang J, Morizono H (2011). GFAP mutations, age at onset, and clinical subtypes in Alexander disease. Neurology.

[9] Eng LF, Ghirnikar RS, Lee YL (2000). Glial fibrillary acidic protein: GFAP-thirty-one years (1969-2000). Neurochem Res.

[10] Yoshida T, Sasaki M, Yoshida M (2011). Nationwide survey of Alexander disease in Japan and proposed new guidelines for diagnosis. J Neurol.

[11] Garbern JY (2005). Leukodystrophies. Neurogenetics.

[12] van der Knaap MS, Naidu S, Breiter SN (2001). Alexander disease: diagnosis with MR imaging. AJNR Am J Neuroradiol.

[13] Adang LA, Sherbini O, Ball L (2017). Revised consensus statement on the preventive and symptomatic care of patients with leukodystrophies. Mol Genet Metab.

[14] Sechi G, Matta M, Deiana GA (2010). Ceftriaxone has a therapeutic role in Alexander disease. Prog Neuropsychopharmacol Biol Psychiatry.

[15] (2021). Alexander disease. https://rarediseases.info.nih.gov/diseases/5774/alexander-disease.

